# Mobile Apps for Caregivers of Older Adults: Quantitative Content Analysis

**DOI:** 10.2196/mhealth.9345

**Published:** 2018-07-30

**Authors:** Molli R Grossman, Deanah Kim Zak, Elizabeth M Zelinski

**Affiliations:** ^1^ Leonard Davis School of Gerontology University of Southern California Los Angeles, CA United States

**Keywords:** mobile apps, aged, elderly, caregivers, family caregivers, carers, adult children, quality of life, dementia

## Abstract

**Background:**

Informal caregivers of older adults provide critical support for their loved ones but are subject to negative health outcomes because of burden and stress. Interventions to provide information and resources as well as social and emotional support reduce burden. Mobile apps featuring access to information, assistance with scheduling, and other features can automate support functions inexpensively and conveniently and reach a greater proportion of caregivers than otherwise possible.

**Objective:**

The aim of this study was to identify mobile apps geared towards caregivers of older adults, catalog features, and suggest best practices for adoption based on empirical findings of beneficial interventions in the caregiving literature.

**Methods:**

Search for apps focused on ones catered for caregivers of older adults in Google Play and iTunes, compiling their features, and identifying features reflecting categories of support identified in successful intervention studies to negative caregiver outcomes. Intervention research indicates that provision of information and resources, assistance in practical problem solving, coordinating care among multiple caregivers, and emotional support reduce caregiver burden.

**Results:**

Despite approximately over 200,000 mobile health–related apps, the availability of mobile apps for caregivers is relatively sparse (n=44 apps) as of October 2017. Apps generally addressed specific categories of support, including information and resources, family communication, and caregiver-recipient interactions. Few apps were comprehensive. Only 8 out of 44 (18%) had features that addressed three or more categories. Few apps provided specific stress reduction exercises for caregivers, which is important for reducing burden.

**Conclusions:**

Mobile apps have the potential to provide resources, just­-in­-time information for problem-solving, and stress reduction strategies for caregivers. Many apps offer functions that have been shown to reduce burden and improve health outcomes in caregivers, but few provide emotional support. Using an evidence­-based practice approach, mobile apps for caregivers can provide multiple beneficial support functions. Apps can serve a much larger proportion of this highly underserved population in their mobile form than more traditional means, improving their health and quality of life.

## Introduction

The rapid increase in longevity and the post-World War II baby boom have produced major demographic changes in the United States. The number of adults over the age of 65 in the US is expected to be 89 million by 2050, more than double the number of older adults in the US in 2010 [1]. Critical developments associated with the increase in longevity are the reduction of acute diseases associated with mortality and the rise in chronic diseases as leading causes of disability, and death [1]. Indeed, the majority of adults over 65 years of age have one or more chronic conditions such as arthritis or hypertension. Importantly, the quality of life also declines with an increasing number of chronic conditions. The ability to perform Instrumental Activities of Daily Living (IADL), such as grocery shopping or housework is likely to be affected by chronic disease. As frailty increases, the ability to engage successfully in basic Activities of Daily Living (ADL), such as toileting, feeding, or bathing may also be compromised. This leads to a loss of independence, and a greater reliance on others for assistance in daily tasks [1]. Because the US health care system is designed to focus on acute care (treating curable illness), the responsibility of managing more chronic or long-term conditions typically falls upon family members, who have been called the “backbone” of this type of care [2].

According to the National Alliance for Caregiving (NAC) and the American Association of Retired Persons (AARP) Caregiving in the US 2015 report [3], an estimated 34.2 million adults in the US provided unpaid care to an adult aged 50 years or older in 2014. The majority of these caregivers provided care for a relative, with 49% caring for a parent or a parent-in-law and 10% providing care for a spouse or partner. The top 3 reported reasons for care were (1) old age, (2) Alzheimer disease or another type of dementia, and (3) surgery or wounds. Family caregivers spend, on average, 24.4 hours a week providing care for their loved ones, and this amount of time is almost doubled to 44.6 hours a week for those providing care for a spouse or partner. Much of caregiving efforts are spent assisting with ADLs, and on average, 4.2 out of 7 IADLs. Also, caregivers often engage in other activities on behalf of their care recipients to coordinate care, such as communicating with care providers, and other agencies [3]. Furthermore, a growing number of family caregivers are members of the so-called “sandwich generation,” balancing care for dependent children and aging parents simultaneously, adding to the complexity and stress of care responsibilities [4].

Researchers have thoroughly documented that high demands of caregiving often lead caregivers to experience stress in physical, mental, and social health. This is a phenomenon commonly referred to as caregiver burden. Although there is no medical diagnosis code for caregiver burden, Zarit et al [5] define it as “the extent to which caregivers perceive that caregiving has had an adverse effect on their emotional, social, financial, physical, and spiritual functioning.” Indeed, as a meta-analysis revealed, in comparison to noncaregivers, caregivers fare worse across 5 indicators of health, including depression, stress, subjective well-being, self-efficacy, and physical health [6]. Risk factors for experiencing caregiver burden include being female, having low education, living with the care recipient, higher number of hours spent providing care, depression, social isolation, financial stress, and the lack of choice in being a caregiver [3,7]. Unfortunately, the amount of caregiver burden continues to rise with the aging population, as individuals are living longer but not necessarily healthier, as evidenced by the continued prevalence of chronic disease [8].

For those providing care for individuals with dementia, the risk of caregiver burden is especially high. Caregivers of individuals diagnosed with moderate to severe dementia, with the inability to perform most IADLs and the presence of behavioral disturbances, tend to experience higher levels of caregiver burden [9]. Higher caregiver burden correlated with dementia severity is seen more in women and older caregivers [10]. In caregivers of individuals with dementia, greater psychological distress, including depression, anxiety, and hostility, tends to occur with increased caregiver burden [11].

One theoretical framework that has played a particularly prominent role in shaping the development of interventions is the stress process framework [12]. The stress process framework combines prior research by Pearlin et al [13] and the transactional model of stress and coping [14]. The theoretical framework asserts that factors in the care context can influence the types of stressors that caregivers tend to experience, the way they appraise and cope with that stress, and caregiving outcomes. The stress framework has been extremely influential in the design of caregiver support services in several ways. First, it demonstrated that caregiver stress involves more than the burden of providing physical care. Instead, as the caregiver experience is influenced by variables across multiple domains, it is not likely that one single intervention will effectively reduce caregiver stress. Thus, the stress framework underscored the need for programs to target multiple domains for intervention. It also led to the development of a wide range of psychosocial interventions. Before the stress process framework, the primary focus of caregiver support programs was providing respite or chore services, which proved to be relatively ineffective on their own [15]. However, the stress process framework has led to a vast increase in psychoeducational programs, two of which are the Savvy Caregiver Program [16] and Powerful Tools for Caregivers [17]. Both of these programs are influenced by the stress and coping framework [14] and have been identified as best practice models for caregiver intervention.

Many intervention studies have focused on reducing caregiver burden among caregivers of older adults with dementia (ie, those with highest burden levels). Interventions that have been found to be most successful have provided them with information about dementia, trained practical problem-solving skills, improved family communication and other social support, and increased caregivers’ sense of self-efficacy [18,19]. Recent studies have delivered such interventions, grounded in stress and coping theory, using forms of remote communication. For example, in the Miami REACH (Resources for Enhancing Alzheimer’s Caregiver Health) program, Cuban American caregivers who were provided with in-person family therapy, access to information databases, and conference calls in conjunction with a Computer-Telephone Integrated System. For example, telephones with monitors that allowed remote visual communication with therapists, showed the most significant reduction in caregiver depression after 6 months. Benefits were sustained at 18 months relative to conventional family therapy or only minimal support [20]. Bank et al [21] also demonstrated the generalization of telephone support groups for ethnically diverse caregivers of individuals with dementia, with similar patterns of benefit from the different features of the intervention.

Although there have been some mixed findings, Web-based caregiving programs have strong potential to increase access to social support from other caregivers and new social contacts, access to relevant information, and support from care professionals. These types of programs have also been shown to lead to improvements in coping skills, confidence, and self-efficacy and significant reductions in caregiver depression, anxiety, stress, and strain [22-26]. A recent review study on Web-based technologies for caregivers of individuals with chronic illness supported these findings that internet-based interventions can improve mental health, physical health, and general caregiving outcomes [27].

The ubiquity of mobile technology also has great potential for reducing caregiver burden. Technology already plays a pivotal role in many aspects of everyday life. According to Nielsen, 71.4% of adults living in the US own a mobile phone [28], and 89% of mobile media time is spent using a mobile app [29]. TechCrunch reported that many users believe apps to be more convenient (55%), faster (48%), and easier to browse (40%) than mobile websites [30]. Also, whereas adoption of traditional computer use has declined or stalled, the adoption of mobile devices such as smartphones continues to grow among Americans over 50 years of age. Over half of this group now owns a smartphone, and this number continues to grow [31].

Mobile app programs are easily accessible, generally inexpensive, and provide a repository for information, which they can integrate from multiple sources [32]. This makes them promising tools for assisting with important health-related activities. For example, Wang et al [33] found that smartphone interventions were effective in helping individuals manage chronic diseases including diabetes, obesity, depression, and cancer. With the help of mobile app programs, patients with chronic conditions participated in their health management more effectively, felt more secure in the knowledge that their illnesses were closely monitored, and felt more connected to their doctors. Although we could find no published study examining the effect of app programs on reducing caregiver burden, it is very likely that mobile technology has vast potential to support caregiving by providing convenient tools and resources to coordinate the demanding tasks and the complex networks of relationships involved in caring for others. Also, Schulz et al [34] found that caregivers are willing to pay for technologies to help their loved ones but unwilling to pay significant amounts, making inexpensive mobile apps an acceptable solution.

In an online survey of 1000 technology using caregivers conducted by the NAC and United Healthcare [35], 7 in 10 respondents reported they would be somewhat or very receptive to using a smartphone for apps to help them with caregiving (69%). Younger caregivers were twice as likely to report being very receptive (43% versus 21% of caregivers 50 years of age or older) to using smartphone apps to help with caregiving needs. Also, those employed full time are more receptive than caregivers who are not employed to using a smartphone to help with caregiving (78% versus 57% very or somewhat receptive), even when controlling for the caregiver’s age. A larger proportion of medium- to high-burden caregivers report being very receptive to using caregiving apps (34%) relative to low-burden caregivers (25%), likely reflecting their greater need for assistance. Furthermore, a recent NAC report featuring results from a roundtable of experts from government, Silicon Valley entrepreneurs, caregiving advocates, and researchers identifies the need for and recommends the development of mobile technology to better support family caregiving [36].

It is estimated that the number of mobile health (mHealth) apps available to consumers exceeds 259,000 [37,38]. A small subset of these apps is designed to assist family caregivers with the specific challenges associated with providing care to older adults with dementia and other chronic diseases. We surveyed and reviewed the types of apps available for caregivers of older adults in October 2017. We identified these apps to determine whether they reflected aspects of support shown to be the most effective in the caregiving literature and could in principle reduce levels of burden, providing much needed, uniquely accessible support to this valuable population.

## Methods

During October 2017, apps in English that self-identified or advertised themselves as tools or aids for caregiving were identified in the iTunes App Store and Google Play, the most prevalent operating systems in use by smartphones, by searching the keywords “caregiving,” “caregiver,” and “elder care.” The internet was also used to identify caregiving apps using the search phrase, “caregiving apps,” in Google Search. Only apps that specifically addressed family or informal caregivers of older adults were included.

Apps developed for professional caregiving provider organizations or apps that help locate professional caregiving services were excluded. Apps that were designed for those living with specific conditions or health issues (eg, stroke or cancer patients) were also excluded from this study, but it is important to acknowledge that these may also be used by caregivers. All apps were classified on their platform availability. This included the Apple iOS or Android and both phone and tablet, cost, and features as described on their iTunes App store or Google Play store page.

## Results

### Census of Relevant Apps

Relative to general mHealth apps, availability of mobile apps geared towards caregivers of older adults is relatively sparse with 44 apps as of October 2017. Nevertheless, this number has been steadily on the rise. Existing caregiving apps generally addressed specific aspects of the caregiving experience. Few apps were comprehensive, with only 7/44 (16%) apps with features that addressed 3 functions, and 1 (2%) app addressing 4 or more (Table 1).

### Content Analysis

The 44 app programs (Multimedia Appendix 1) were categorized separately by two coders (authors MG and DZ) and the differences were resolved by discussion. The categories were not mutually exclusive (Table 2). They represented functions served by successful caregiver burden reduction interventions: (1) information and resources, (2) practical problem-solving involving behavioral solutions, medication management, safety, and personal health record tracking, (3) memory aids, (4) family communication, including coordinating care, calendars for appointments and sharing, medical and emergency contact lists, ability to share important information, photos, and messages among caregivers and family members, and (5) caregiver support (ie, care for the caregiver), and comprehensive apps that integrated multiple functions [18,19]. Of the 44 apps, 22 (50%) were specifically designed for caregivers of individuals with dementia.

#### Information and Resources

Fifteen (34%) apps met criteria for providing caregivers with *Information and Resources*. These apps provide medical information and expert advice on topics in aging or elder care. Some include searchable databases on a wide variety of medical conditions, with features that include videos, symptom tracking, personalized reports, and first aid essentials. Of these 15, 9 (60%) apps were designed specifically for caregivers of individuals with Alzheimer disease or other forms of dementia, offering information and helpful solutions for difficult dementia-related behaviors. The remaining 6 (40%) were for caregivers of older adults, providing important information on more general eldercare topics.

#### Practical Problem Solving

A total of 21 app programs addressed practical problem-solving needs that many caregivers share. Practical problem solving was defined as addressing medication management, safety, personal health record tracking, or behavioral solutions. Three of the 21 (14%) apps contained tools for *Medication Management*. Common features for these apps include medication schedule and reminder programs, missed dose alerts, refill reminders, searchable drug databases with drug information such as dosage, indication, side effects, and drug interactions. While many other medication management apps exist, they are not geared toward caregivers of older adults, and thus were excluded from the search.

One serious concern for caregivers of family members with Alzheimer disease or other types of dementia is wandering by the care recipient. For example, 60% of the individuals with dementia will wander, and this can lead to dangerous consequences [39,40]. Five (24%) apps contained tools to address the *Safety* of loved ones by monitoring their movements. Two (10%) of these apps used GPS to inform caregivers of the location of their care recipients and relied on their having a GPS-enabled phone on their person. Of these, 1 (5%) app was available in iOS only and the other was available in both iOS, and Android. Both were free, and 1 was designed to improve the autonomy of individuals with Alzheimer disease in the early stages of the disease. Walking has been found to be beneficial in the early stages of this disease, and these apps might make it more likely for individuals with Alzheimer disease to go for walks on their own, supporting an also critical sense of autonomy. The remaining 3 (14%) apps featured wearable technology or home sensors at an added cost that allow family caregivers to monitor their loved ones’ whereabouts. These apps were designed specifically as a tool for caregivers of persons with dementia.

**Table 1:** Number of functions associated with caregiving apps (N=44).

Number of functions	n (%)
1	25 (57)
2	11 (25)
3	7 (16)
4	1 (2)

**Table 2:** Apps meeting the criteria for each category of features (N=44). Note that some apps meet the criteria for more than one category.

Category	Examples and features	Platforms, n (%)			
		iOS	Android	Both	Total
Information and resources	Tips and advice; information about dementia, other diseases; searchable databases; videos; symptom tracking	4 (9)	3 (7)	8 (18)	15 (44)
Family communication	Care coordination among family; instant messaging; calendar sharing; to-do lists	3 (7)	2 (5)	10 (23)	15 (44)
Memory aids	Activities for care recipient; conversation starters; tools for memory support and reminiscence	5 (11)	4 (9)	3 (7)	12 (27)
Care for caregiver	Support/chat groups Burden assessments; words of encouragement	2 (5)	4 (9)	4 (9)	10 (23)
Behavior solutions	Tips and information to manage problem behaviors (eg, agitation, wandering)	3 (7)	2 (5)	3 (7)	8 (18)
Medication management	Medication reminders; dosage information; drug-interaction databases	—	—	3 (7)	3 (7)
Safety	GPS^a^/motion sensor tracking; automated check-in calls; alarms and reminders	1 (2)	1 (2)	3 (7)	5 (11)
Personal health record tracking	Doctors’ appointment reminders	1 (2)	—	4 (9)	5 (11)

^a^GPS: Global Positioning System.

*Personal Health Record Tracking* apps allow caregivers to collect, track and share past and current health information about a care recipient. They often accommodate multiple user profiles with emergency contact information, health insurance, doctors' contact information, and reminders for doctors’ appointments, and other upcoming medical appointments on a calendar. Five of the 21 (24%) practical problem-solving apps were identified that provided specific health record tracking tools to assist caregivers with managing their loved one’s health.

In addition, 8 apps (38%) provided suggested solutions for dealing with behavioral problems that can arise in the care recipient. All of these apps were specifically designed for those caring for individuals with Alzheimer disease or other forms of dementia, as behavioral disturbances are often present in the middle and later stages of the disease and can be challenging for family members to handle. Examples of behavioral solutions or tips provided by these apps include how to address sleep disturbances, delusions, hallucinations, wandering, and catastrophic responses to stressors later in the day possibly associated with light changes towards sundown (“sundowning”).

#### Family Communication

Our search yielded 15 apps that facilitate better communication for coordinating care among family members. These apps were designed as communication tools to allow family, friends, neighbors, and colleagues to coordinate care needs for the care recipient. The apps allow caregivers to create profiles of their loved one containing health information pertinent to care, such as medical condition, medication list, and emergency contacts. An important feature of many of these apps is a shared calendar to coordinate the efforts of multiple family members or caregivers to assure recipient’s needs are addressed. With features such as instant messaging, sharing of photos and videos and other updates, they also provide a much-needed support network for caregivers. Only 2/15 (13%) of these apps were specifically designed for caregivers of persons with dementia, though the others were all designed for elder care.

#### Memory Aids

Importantly, some apps aim to reduce caregiver burden by stimulating or supporting the needs of the care recipient. There has been a recent influx of care recipient focused *Memory Aid* apps (currently numbering 12 apps), designed to assist individuals with Alzheimer disease and other types of dementia by supporting enhanced cognitive function, communication, and quality of life. Two of these 12 (17%) apps serve as tools for memory assistance, providing cues and reminders for a list of tasks for people with dementia, traumatic brain injury, or other memory disorders. Nine (75%) apps aim to improve the quality of life for people with dementia and their caregivers through shared activities such as making an interactive life storybook, or providing story starters, memory games, and music and videos. Additionally, 1 (8%) app provides a picture-based communication tool for caregivers of individuals with little to no verbal communication ability, including those in the later stages of dementia.

#### Care for Caregiver

Caregivers are providing a significant service managing their loved ones’ health, but occasionally the stressful nature of this role can become detrimental to their own health. A total of 10/44 (23%) apps contained components to address caring for the caregiver, in the sense of providing emotional or social support or forms of stress relief, and respite. Apps in this category contain features such as chats or app-based support groups or social networks, assessments to track stress and burden level to be aware of one’s condition, suggestions for supporting one’s own health and quality of life and encouraging words, and advice from other caregivers.

#### Comprehensive

*Comprehensive* app programs combine some of the functions described thus far including: symptom tracking and journaling, medication management with refill reminders, calendars for appointments and coordinating care, medical record profiles with emergency contact lists, and features that enable sharing of information, photos, messages among caregivers and other family members. While no apps in our October 2017 search addressed all empirically-derived components of necessary caregiver support, 1/44 (2%) app was found that addressed at least half (4 out of 8) of these components and thus was deemed comprehensive in nature.

The *Balance: For Alzheimer Caregivers* app allows caregivers to coordinate care among multiple family members by tracking physical, behavioral, and emotional changes in the care recipient and sharing the information with other caregivers and doctors in real time using a chat feature. It also enables multiple caregivers to manage caregiving tasks and provides a newsfeed with the latest Alzheimer disease and caregiving findings and information. Additionally, the app includes a one-click button to connect to a free 24-hour helpline, available in a range of languages, and a “doctor diary” which caregivers can use to communicate with doctors about changes in symptoms and behaviors in “real time.” This app is created by RiverSpring Health, a senior health care organization, and CaringKind, a dementia non-profit which was formally the New York City chapter of the Alzheimer’s Association. CaringKind professionals manage the 24-hour helpline, lending support to its credibility (Note that these are the features as described in 2017, and apps frequently undergo changes and updates.)

### Cost and Platform Availability

The apps meeting our search criteria varied in cost and platform availability. Most were available for free, although some required or encouraged supplemental in-app purchases. Of those that did cost to download, the fee ranged from US $0.99 to US $29.99 making them relatively affordable. The only exceptions were the safety apps requiring additional GPS or sensor technologies. While the apps themselves were free, 2 required wearable sensors and hardware kits that start at US $249.99, and 1 offered home-based sensor technology at subscription rate ranging from approximately US $60 to US $170 per month, depending on the number of sensors needed.

Most apps (23/44, 52%) were available for both iOS and Android devices. However, of those available in only 1 platform, slightly more were available for download for iOS than for Android devices.

## Discussion

### Principal Findings

In summary, over the last few years, there has been growth in the number of mobile apps targeted at caregivers of older adults and designed to help them manage caregiving responsibilities and reduce levels of burden. However, in comparison to the large number of mHealth apps and caregiving apps available more generally, there are still relatively few apps catering specifically to the unique needs of caregivers of older adults. As the baby boomer generation is aging, this country is bracing to deal with the largest proportion of older adults our population has ever seen. For the first time in the US and globally, adults 60 years of age and older will soon outnumber children aged 5 years and under. [41]. The primary responsibility to care for this growing number of older adults, along with their chronic health conditions, is poised to fall upon family caregivers. However, because of other simultaneously occurring demographic changes, such as lower birth rate and greater geographic dispersion of family members, the number of available family caregivers is decreasing, leaving an unfortunate imbalance between those available to provide care and those who need it [42].

Alzheimer disease and other types of dementia are projected to increase drastically with the aging of the population. Currently, Alzheimer disease affects over 5 million Americans, and it is projected that this number will triple by 2050 [43,44]. The majority of individuals with dementia are cared for at home by family members. Fortunately, our review showed an increase in recent development of mobile apps targeted at Alzheimer disease and other forms of dementia, with a particularly large number of apps addressing the domains of information and resources for caregivers and memory aids and enrichment for care recipients. This development is encouraging, and it is important that apps in this area continue to grow, as there is still no cure for Alzheimer disease, an illness that takes a huge toll on both the caregiver and recipient. For this reason, it is also a positive development to see apps directed at the care recipient as well as the caregiver. For example, certain apps were designed to help those with dementia maintain feelings of autonomy and independence for as long as possible. Others aim to foster improved interaction and communication between the caregiver and care recipient, a relationship that can sometimes become tense on both sides. A focus on individuals with dementia and their family members as care partners, rather than as patient and caregiver, is promising and in line with research on the increasingly recognized value of person-centered care [45,46].

Another finding revealed by this study is that caregiving apps still have room for improvement regarding their comprehensiveness. For instance, 25/44 (57%) of the apps surveyed provided only 1 service to caregivers. Only 8 (17%) of the 44 apps provided 3 or more services. Caregivers are already balancing various daily tasks, medical appointments, jobs, and more. Thus, it might be ideal to combine as many useful features as possible into 1 easy-to-use app. The goal of mobile apps to support caregivers should be to reduce their load to help their productivity rather than hinder it. Also, as mentioned previously, the stress process framework suggests that caregivers experience stress in a larger context and targeting just one problem or fulfilling a single need will likely be less effective than an intervention targeting multiple needs [15].

In addition, most of the caregiving apps currently on the market do not seem to have been developed with the guidance of caregiving researchers. Therefore, there may be gaps between app features and empirical findings regarding caregiver burden and effective means of intervention. Future developers should collaborate with academic researchers to ensure that their apps are designed with the current empirical evidence in mind. For example, one crucial area particularly overlooked by most existing apps is caring for the caregiver. While 10 (23%) apps provided some form of limited caregiver support, most of the apps focused on providing solutions to concrete problems (eg, tracking a wandering loved one, dealing with problematic behaviors, communicating with other family members, or keeping a health record). Though these are pressing needs, there is still an imperative demand for resources focusing on supporting the caregiver’s emotional well-being, including coping with stress, anxiety, and depression. Our search yielded many general meditation and stress relief apps, but none were found to be directed toward caregivers of older adults and their unique stressors and experiences.

### Recommendations for Outreach and Research

Family caregivers are providing an indispensable service to society, saving the health care system billions of dollars annually. Often, they do so to the detriment of their health and well-being. Furthermore, studies suggest that when the caregivers’ mental health is strained, their care recipients may suffer as well, regarding worsened health and shortened longevity [47]. Therefore, it is vital to invest time and energy into developing technology with the potential to vastly improve the lives of caregivers and their loved ones receiving care.

Importantly, though they may be the ones who have the most to gain from this technology, family caregivers have been found to be less likely to use mobile apps than the general public [48]. This imbalance suggests that greater efforts need to be made to reach this population and show them the value of this burgeoning technology. Caregivers need to be made aware that these types of resources exist at their fingertips. Another potential barrier is that many family caregivers do not identify as care partners, sometimes for many years, and therefore may not search for resources like mobile apps. Policymakers and insurance providers should consider policies promoting the use of mHealth apps that have been shown to be effective at improving health outcomes via subsidies or other incentives [48]. Health professionals might also consider providing their patients and family caregivers with information on mobile apps available to them as a means of additional support, and can guide them in terms of selecting apps with evidence-based content.

Models of technology acceptance suggest that users, including older adults, are willing to invest in new technologies if they perceive enough usefulness and ease of use [49-51]. Therefore, it is critical that apps reflect features that users find most beneficial. It is also critical that apps focus on being intuitive and easy to operate. One study examined mHealth app use among research participants, including those managing diabetes, depression, or caregiving, and found that many highly rated apps were still too difficult for participants to use [52]. Furthermore, caregivers are a group of people for whom time is typically strained, thus underscoring the value of simple, clear, and easy to operate technologies and interfaces.

For reasons such as these, user experience studies are needed to customize apps to the needs, desires, and abilities of the targeted users. For instance, it may prove useful to develop adaptive material for caregivers whose care recipients are experiencing different stages of dementia. A strategy that works to calm or stir memories in a person with dementia may work well initially but lose its effectiveness as the disease progresses. Dementia is a progressive disease, meaning that symptoms will change and worsen over time. Therefore, a one-size-fits-all approach may not be appropriate for dementia caregivers, at least not without addressing the diversity of caregivers and the evolving needs of their care recipients. Additionally, many caregivers of older adults are over 65 years of age themselves, caring for a spouse or other loved one. As such, app designers should involve older adults in the design process as much as possible, as opposed to simply testing with younger demographics. Apps that are user-friendly for younger adults may not be user-friendly for older users.

Another consideration that will be important as the field of mHealth continues to move forward is that of security and privacy, for example with video monitoring of care recipients. Of course, with the advent of these new technologies comes the great potential for increased supervision, safety, and support. However, these developments also give rise to important questions regarding the invasion of privacy and security of information [53,54]. Privacy concerns may be especially relevant to older adults, who voice the most concern over the privacy and security of their information online (AARP, 2017). To address these concerns, it has been argued that mHealth apps especially need to focus on standard app development guidelines and security authentication measures, such as device and app passwords, strong encryption mechanisms, and informative privacy policies [55].

Another question worth considering is whether 1 app can, or should, actually “do it all.” While it is our opinion that simplicity, by way of fewer apps, will be preferred by caregivers, we must recognize that this may not be the case. Perhaps well-developed apps, each with a different focus, will be equally useful and preferred for the sake of compartmentalization. However, during our review, 6 caregiving apps that were included in an early count in December 2016 disappeared by October 2017, each of which had provided a singular service. This turnover pattern might provide support for the futility and limited appeal of single function apps for caregivers. Lastly, it is essential to take into account the frequency and duration of app use. If an app’s sole purpose is to provide information and resources, will caregivers continue usage after they have acquired the specific information they sought? For this reason, an app offering multiple features may be favored by caregivers and used more regularly.

### Conclusions

In conclusion, the findings discussed in this paper should inform future work to develop more evidence-based, comprehensive apps to support caregivers’ needs and reduce caregiver burden. Apps may be an easy, accessible, and relatively inexpensive way to help this population manage and navigate the day-to-day challenges of the caregiving role, especially for those who do not have the time or means to seek out in-person support. More research is needed on effective mobile app interventions and resources for caregivers, including piloting with and surveying caregivers themselves to see how this technology can best suit their needs and preferences. It is extremely encouraging to see the number of relatively new apps supporting family caregivers of older adults. However, taking into account the input of caregivers themselves and incorporating the most up-to-date evidence emerging from research will likely be critical to the success and effectiveness of these apps. With the rapid growth of mobile phone use in our population and the simultaneous growing number of older adults, a golden opportunity exists to utilize mobile phone technologies to help manage their caregiving needs.

## Appendix

Multimedia Appendix 1 Catalog of caregiving apps and their features.

## Abbreviations

AARP: American Association of Retired Persons
ADL: Activities of Daily Living
GPS: Global Positioning System
IADL: Instrumental Activities of Daily Living
mHealth: mobile health
NAC: National Alliance for Caregiving

## References

[1] (2013). The state of aging and health in America 2013. Centers for Disease Control and Prevention.

[2] Kane R (2005). Meeting the challenge of chronic illness.

[3] National AFC, AARP (2015). Caregiving in the US; NAC and AARP Public Publishing Institute. AARP.

[4] Boyczuk AM, Fletcher PC (2015). The Ebbs and Flows: Stresses of Sandwich Generation Caregivers. J Adult Dev.

[5] Zarit SH, Todd PA, Zarit JM (1986). Subjective Burden of Husbands and Wives as Caregivers: A Longitudinal Study. The Gerontologist.

[6] Pinquart M, Sörensen S (2003). Differences between caregivers and noncaregivers in psychological health and physical health: A meta-analysis. Psychology and Aging.

[7] Adelman RD, Tmanova LL, Delgado D, Dion S, Lachs MS (2014). Caregiver burden: a clinical review. JAMA.

[8] Crimmins EM, Beltrán-Sánchez H (2011). Mortality and morbidity trends: is there compression of morbidity?. J Gerontol B Psychol Sci Soc Sci.

[9] Hall D, Wilkerson J, Lovato J, Sink K, Chamberlain D, Alli R, Clarke P, Rogers S, Villalba J, Williams J, Shaw E (2014). Variables Associated with High Caregiver Stress in Patients with Mild Cognitive Impairment or Alzheimer's Disease: Implications for Providers in a Co-Located Memory Assessment Clinic. Journal of Mental Health Counseling.

[10] Ziello A, Pastore F, Fasanaro AM, Poderico C, Iavarone A (2014). Caregiver burden and coping strategies in caregivers of patients with Alzheimer&rsquo;s disease. NDT.

[11] Razani J, Corona R, Quilici J, Matevosyan A, Funes C, Larco A, Miloyan B, Avila J, Chung J, Goldberg H, Lu P (2014). The Effects of Declining Functional Abilities in Dementia Patients and Increases in Psychological Distress on Caregiver Burden Over a One-Year Period. Clinical Gerontologist.

[12] Pearlin LI, Mullan JT, Semple SJ, Skaff MM (1990). Caregiving and the stress process: an overview of concepts and their measures. Gerontologist.

[13] Pearlin L, Menaghan E, Lieberman M, Mullan J (1981). The Stress Process. Journal of Health and Social Behavior.

[14] Lazarus R, Folkman S, Gentry WD (1984). Coping and Adaptation. The Handbook of Behavioral Medicine.

[15] Bengtson VL, Settersten Jr R, Montgomery, Kwak, Kosloski (2016). Theories Guiding Support Services for Family Caregivers. Handbook of Theories of Aging.

[16] Cleland M, Schmall VL, Studervant M, Congleton L, Kirkbride K, McFalls J, Shannon K, Sponsler V, Turner H (2006). The caregiver helpbook: Powerful tools for caregivers.

[17] Hepburn KW, Lewis M, Sherman CW, Tornatore J (2003). The savvy caregiver program: developing and testing a transportable dementia family caregiver training program. Gerontologist.

[18] Ostwald SK, Hepburn KW, Caron W, Burns T, Mantell R (1999). Reducing Caregiver Burden: A Randomized Psychoeducational Intervention for Caregivers of Persons With Dementia. The Gerontologist.

[19] Chen H, Huang M, Yeh Y, Huang W, Chen C (2014). Effectiveness of coping strategies intervention on caregiver burden among caregivers of elderly patients with dementia. Psychogeriatrics.

[20] Eisdorfer C, Czaja SJ, Loewenstein DA, Rubert MP, Arguelles S, Mitrani VB, Szapocznik J (2003). The Effect of a Family Therapy and Technology-Based Intervention on Caregiver Depression. The Gerontologist.

[21] Bank AL, Argüelles S, Rubert M, Eisdorfer C, Czaja SJ (2006). The value of telephone support groups among ethnically diverse caregivers of persons with dementia. Gerontologist.

[22] Topo P (2008). Technology Studies to Meet the Needs of People With Dementia and Their Caregivers: A Literature Review. Journal of Applied Gerontology.

[23] Chiu T, Marziali E, Colantonio A, Carswell A, Gruneir M, Tang M, Eysenbach G (2009). Internet-based caregiver support for Chinese Canadians taking care of a family member with alzheimer disease and related dementia. Can J Aging.

[24] Godwin KM, Mills WL, Anderson JA, Kunik ME (2013). Technology-driven interventions for caregivers of persons with dementia: a systematic review. Am J Alzheimers Dis Other Demen.

[25] Boots L, Vugt M, Knippenberg R, Kempen G, Verhey F (2014). A systematic review of internetbased supportive interventions for caregivers of patients with dementia. International Jounal of Geriatric Psychiatry.

[26] Lundberg S (2014). The results from a two-year case study of an information and communication technology support system for family caregivers. Disabil Rehabil Assist Technol.

[27] Ploeg J, Markle-Reid M, Valaitis R, McAiney C, Duggleby W, Bartholomew A, Sherifali D (2017). Web-based interventions to improve mental health, general caregiving outcomes, and general health for informal caregivers of adults with chronic conditions living in the community: Rapid evidence review. Journal of Medical Internet Research.

[28] (2014). Mobile millennials: Over 85% of generation y owns smartphones.

[29] (2013). An era of growth: The cross-platform report Q4.

[30] Perez S (2013). Users have low tolerance for buggy apps- only 16% will try a failing app more than twice.

[31] Anderson G (2016). Technology trends among mid-life and older Americans. AARP Research.

[32] Dayer L, Heldenbrand S, Anderson P, Gubbins PO, Martin BC (2013). Smartphone medication adherence apps: Potential benefits to patients and providers. Journal of the American Pharmacists Association.

[33] Wang J, Wang Y, Wei C, Yao NA, Yuan A, Shan Y, Yuan C (2014). Smartphone interventions for long-term health management of chronic diseases: an integrative review. Telemed J E Health.

[34] National Alliance for Caregiving and United Healthcare.

[35] Schulz R, Beach SR, Matthews JT, Courtney K, De VDA, Mecca LP (2016). Caregivers' Willingness to Pay for Technologies to Support Caregiving. Gerontologist.

[36] Adler R, Mehta R (2014). Catalyzing technology to support family caregiving. National Alliance for Caregiving.

[37] Murray A, Lyle J (2015). Patient adoption of mHealth: Use, evidence and remaining barriers of mainstream acceptance. IMS Institute for Healthcare Informatics.

[38] Research2Guidance (2016). mHealth App Developer Economics.

[39] Alzheimer's Association.

[40] Gergerich E, Davis L (2017). Silver Alerts: A Notification System for Communities with Missing Adults. J Gerontol Soc Work.

[41] (2015). World Health Organization.

[42] Wolff JL, Feder J, Schulz R (2016). Supporting Family Caregivers of Older Americans. N Engl J Med.

[43] Hebert LE, Weuve J, Scherr PA, Evans DA (2013). Alzheimer disease in the United States (2010-2050) estimated using the 2010 census. Neurology.

[44] Hung Y, Kadziola Z, Brnabic AJ, Yeh J, Fuh J, Hwang J, Montgomery W (2016). The epidemiology and burden of Alzheimer's disease in Taiwan utilizing data from the National Health Insurance Research Database. Clinicoecon Outcomes Res.

[45] Kogan AC, Wilber K, Mosqueda L (2016). Person-Centered Care for Older Adults with Chronic Conditions and Functional Impairment: A Systematic Literature Review. J Am Geriatr Soc.

[46] Love K, Pinkowitz J (2013). Person-centered care for people with dementia: A theoretical and conceptual framework. Generations.

[47] Lwi SJ, Ford BQ, Casey JJ, Miller BL, Levenson RW (2017). Poor caregiver mental health predicts mortality of patients with neurodegenerative disease. Proc Natl Acad Sci U S A.

[48] Kim H, Paige PM, Bhuyan SS, Bhuyan SS (2017). Seeking Medical Information Using Mobile Apps and the Internet: Are Family Caregivers Different from the General Public?. J Med Syst.

[49] Eggermont S, Vandebosch H, Steyaert S (2006). Towards the desired future of the elderly and ICT: policy recommendations based on a dialogue with senior citizens. Poiesis Prax.

[50] Ijsselsteijn W, Nap H, de Kort Y, Poels K (2007). Digital game design for elderly users.

[51] Melenhorst A (2002). Adopting communication technology in later life: the decisive role of benefits (PhD).

[52] Sarkar U, Gourley GI, Lyles CR, Tieu L, Clarity C, Newmark L, Singh K, Bates DW (2016). Usability of Commercially Available Mobile Applications for Diverse Patients. J Gen Intern Med.

[53] Faudree B, Ford M (2013). CIO Journal.

[54] Kharrazi H, Chisholm R, VanNasdale D, Thompson B (2012). Mobile personal health records: an evaluation of features and functionality. Int J Med Inform.

[55] Adhikari R, Richards D, Scott K (2014). Security and privacy issues related to the use of mobile health apps.

