# Supplementary material for: Mobile Apps for Caregivers of Older Adults: Quantitative Content Analysis
Source: JMIR Mhealth Uhealth. 2018 Jul 30;6(7):e162. doi: 10.2196/mhealth.9345 (PMC6090169; doi:10.2196/mhealth.9345)
Supplement: Multimedia Appendix 1 [file mhealth_v6i7e162_app1.pdf]

Appendix.

Appendix Table 1. Catalog of Caregiving Apps and Their Features.

			Features							
Applications	Platform (IOS or Android)	Cost	Information and Resources	Behavioral Solutions	Med Management	Safety	Personal Health Record Tracking	Family Communication	Memory Aids	Care for Caregiver
Ahlov Family Reminders	I	Free trial ($19.99/ month)						X		
Alzheimer’s (and Other Dementias) Daily Companion	I/A	Free/ Free	X	X						
Alzheimer’s and Dementia Tips for Families	I	Free (in app $)	X	X						
Alzheimer's Caregiver Buddy	A	Free	X	X						X
Alzheimer’s Story Starters	I/A	Free/ Free							X	
Alzium Caregiver	I/A	Free/ Free				X		X		
Alzminder Lite	A	Free							X	
Balance: for Alzheimer's Caregiving	I	$0.99	X	X			X	X		
Caregivers in the Community (CINC)	I/A	Free/ Free	X							X
CaregivingApp		Free/								

Caregiver	I/A	Free			X			X		
Caring Village	I/A	Free/ Free						X		
Carely	I/A	Free/ Free						X		
CareZapp	I/A	Free/ Free (*)				X	X	X		
Carezone	I/A	Free/ Free			X		X	X		
Care3	I/A	Free/ Free						X		
Care4Dementia	I/A	Free/ Free	X	X						X
Cue You: Memory Help	I	$29.99							X	
Dementia Caregiver Application	I/A	Free/ Free	X	X						
Dementia Caregiver Solutions	I	$2.99	X	X						X
Dementia Guide Expert	I/A	Free/ Free	X							
Dementia Support	I/A	Free/ Free	X							X
Elderly Care (SCI)	I/A	Free/ Free	X							X
Elderly Care Tips	A	Free	X							X
GreyMatters: Reaching Beyond Dementia	I	Free (in-app $)							X	
HABC	A	Free	X	X						X
ICMed	I/A	Free/ Free					X	X		
InfoSAGE	I/A	Free/ Free						X		
It’s Done!	I/A	$2.99							X	

Kinto: Care Better	I/A	Free/ Free	X		X			X		
Lotsa Helping Hands	I	Free						X		
Making Care Easier	A	Free						X		
MemBook	A	Free							X	
Memories Alive	I	Free							X	
Memory Box	I/A	Free/ Free							X	
Mid-stage Alzheimer's Cards	A	Free							X	
MIND App for Alzheimer's, Parkinson's & …	I	Free							X	
MindMate	I	Free (in-app $)							X	
My Medical	I/A	$4.99					X			
SafeWander	I	Free				X				
SmallCircles	I/A	Free/ Free								X
Smart Caregiver	A	Free				X		X		
Tender Loving ElderCare - Family Caregiver's Guide	I	Free	X							X
Tweri Alzheimer's Caregiving Tool	I/A	Free/ Free				X				
What's Next Lite/ What's Next (Visual Prompts)	A	Free (lite); $1.49 (full)							X	

* optional monthly subscription, [a]”in app $” means optional in-app purchases
